# The association of *RAN* and* RANBP2* gene polymerphisms with Wilms tumor risk in Chinese children

**DOI:** 10.7150/jca.36651

**Published:** 2020-01-01

**Authors:** Xiaokai Huang, Jie Zhao, Wen Fu, Jinhong Zhu, Susu Lou, Xiaoqian Tian, Shanshan Chen, Jichen Ruan, Jing He, Haixia Zhou

**Affiliations:** 1 Department of Hematology, The Second Affiliated Hospital and Yuying Children's Hospital of Wenzhou Medical University, Wenzhou 325027, Zhejiang, China; 2 Department of Pediatric Surgery, Guangzhou Institute of Pediatrics, Guangzhou Women and Children's Medical Center, Guangzhou Medical University, Guangzhou, Guangdong 510623, China; 3 Department of Clinical Laboratory, Biobank, Harbin Medical University Cancer Hospital, Harbin 150040, Heilongjiang, China

**Keywords:** * RAN*, * RANBP2*, polymorphism, Wilms tumor, susceptibility

## Abstract

Wilms tumor is considered to be the most common renal malignancy among children. RAN, a member of RAS superfamily, and its binding partner RANBP2 are related to the progression of multiple tumors. Nevertheless, the effects of the *RAN* and* RANBP2* gene polymorphisms on the tumorigenesis of Wilms tumor remain unclarified. In this study, three potentially functional polymorphisms (rs56109543 C>T, rs7132224 A>G, and rs14035 C>T) in the *RAN* and one (rs2462788 C>T) in the *RANBP2* were chosen to investigate their association with Wilms tumor susceptibility. Odds ratios (ORs) and 95% confidence intervals (CIs) were applied to assess the association of the selected polymorphisms with Wilms tumor susceptibility. Results shown that *RAN* rs7132224 AG/GG genotypes significantly increased Wilms tumor risk when compared to AA genotype (adjusted OR=1.40, 95% CI=1.01-1.95, *P*=0.047). Carriers of 1-3 risk genotypes have a significantly higher Wilms tumor risk than those without risk genotype (adjusted OR=1.49, 95% CI=1.07-2.07, *P*=0.020). Moreover, stratified analysis indicated that *RAN* rs56109543 CT/TT genotypes, *RAN* rs7132224 AG/GG genotypes and *RANBP2* rs2462788 CT/TT genotypes remarkably increased Wilms tumor susceptibility among the subgroups. Our results indicated that *RAN* and* RANBP2* polymorphisms were associated with Wilms tumor susceptibility in Chinese children. The role of *RAN/RANBP2* in cancers deserves more attention.

## Introduction

Wilms tumor, resulting from deviant cellular proliferation and differentiation, is currently one of the most concerned tumors in children 1, 2. It is also one of the most common solid pediatric tumors, especially before the age of five years 3. This tumor affects 1 in 10000 children and constitutes 8% of pediatric cancers 4. Although Wilms tumor have an overall survival rate of 80%, more than 15% of patients have a poor prognosis despite aggressive retreatment 5. The patients with anaplastic Wilms tumor, relapsed and blastemal-type disease have a very low survival rate 6. Therefore, it is indispensable to find more effective therapies in order to improve the poor prognosis of Wilms tumor.

In recent years, it is gradually recognized that the occurrence and development of Wilms tumor is closely connected to various genetic alterations 7. For instance, aberrant splicing of* WT1*, a pivotal cancer suppressor gene, increased the risk of Wilms tumor 8. *MYCN* amplification enhances promoted the development of Wilms tumor through multiple mechanisms 9. Moreover, numerous gene polymorphisms have been found to predispose to Wilms tumor 10-12, including *WTX* 13, *P53* 14,* CTNNB1* 15, *BARD1* 16, *HACE1* 17, *LIN28B* 18, *XPD* 19, *hOGG1* and *FEN1* gene 20, *KRAS* 21. Although quite a few of predisposing genetic factors have been found, they are far from enough to explicitly explain the genetic pathogenesis of Wilms tumor.

RAN*,* belonging to RAS superfamily, is a small GTP-binding protein, which is composed of 216 amino acids with typical sequence motifs. Previous experiments have demonstrated that RAN was closely related to mitotic process, nucleo-cytoplasmic transport, nuclear transport of proteins, and the formation of nuclear envelope 22-24. RAN is regarded as a decisive molecular switch for chromosome arrangement and mitotic progression. When nucleotide-free RAN binds to Mog1, not only is anaphase of mitosis delayed, but also many mitotic defects appeared 25. RANBP2, recognized as a pleiotropic protein, plays physiological roles in reconciling gene-environment interactions. It consists of several domains, including four RAN-binding domains 26. Both RAN and RANBP2 are involved in mitosis and are associated with a variety of cancers. Thus, we conducted this study to examine the association of *RAN* and* RANBP2* gene polymorphisms with Wilms tumor risk as well as cumulative effects of polymorphisms in the two genes.

## Materials and methods

### Study subjects

This study was authorized by the Institutional Review Board of Guangzhou Women and Children's Medical Center and Yuying Children's Hospital of Wenzhou Medical University. Written informed consents were obtained from all participants signed by their guardians. In this retrospective research, 183 patients diagnosed with Wilms tumor and 603 cancer-free volunteers were recruited (**Supplementary Table 1**). All the included patients were newly diagnosed and had histopathologically verified Wilms tumor. The exclusion criteria included prior chemotherapy or radiation, other types of tumors, and secondary or recurrent tumors. Controls were recruited from children receiving regular physical examination int the designated hospitals during the same period. Age and sex were matched between cases and controls. Both the cases and controls were unrelated ethnic Chinese Han individuals.

### Polymorphisms selection and genotyping

Three potentially functional polymorphisms in the *RAN* (rs56109543 C>T, rs7132224 A>G, rs14035 C>T) and one potentially functional polymorphism in the *RANBP2* (rs2462788 C>T) were chosen in this study. The criteria of SNPs selection and genotyping have been described in our previous study 27.

### Statistical analysis

Chi-squared test was used to compare the demographic variables and the frequency distribution of gene polymorphisms between cases and controls. The goodness-of-fit χ^2^ test was used to estimate the Hardy-Weinberg equilibrium (HWE) in control subjects. Univariate and multivariate logistic regression analyses were conducted. Odds ratios (ORs) and 95% confidence intervals (CIs) were used to assess the association of *RAN/RANBP2* gene polymorphisms with Wilms tumor susceptibility. All of the statistical tests adopted bilateral. Results were recognized as statistically significant if *P*<0.05.

## Results

### Associations between *RAN/RANBP2* polymorphisms and Wilms tumor risk

The whole genotype frequencies of polymorphisms in the* RAN* and* RANBP2* gene were outlined in **Table 1**. It is apparently that rs7132224 AG/GG genotypes carriers were associated with an increased risk of Wilms tumor when compared with AA genotype carriers (adjusted OR=1.40, 95% CI=1.01-1.95, *P*=0.047). However, no significant association was detected between the other three polymorphisms and Wilms tumor susceptibility. Furthermore, higher risk was demonstrated in participants with 1-3 risk genotypes (adjusted OR=1.49, 95% CI=1.07-2.07, *P*=0.020) when compared with those without risk genotype.

### Stratified analysis

As shown in **Table 2** and **Table 3**, the *RAN* rs56109543 CT/TT genotypes (adjusted OR=1.98, 95% CI=1.15-3.38, *P*=0.013) and *RAN* rs7132224 AG/GG genotypes (adjusted OR=2.80, 95% CI=1.64-4.77, *P*=0.0002) increased the risk of Wilms tumor among the children aged ≤18 months. The presence of 1-3 risk genotypes have an increased risk of Wilms tumor in children aged ≤18 months (adjusted OR=2.80, 95% CI=1.64-4.77, *P*=0.0002) and male (adjusted OR=1.57, 95% CI=1.01-2.46, *P*=0.046) when compared with those without risk genotype. In addition, the *RANBP2* gene rs2462788 CT/TT conferred the susceptibility to Wilms tumor in children aged ≤18 months (adjusted OR=2.43, 95% CI=1.15-5.12, *P*=0.020), male (adjusted OR=2.15, 95% CI=1.08-4.25, *P*=0.029) and subgroup with clinical stage I+II Wilms tumor (adjusted OR=2.77, 95% CI=1.41-5.46, *P*=0.003).

### Haplotype analysis

As presented in **Table 4**, compared to haplotype TTT, the following four haplotypes significant increased the risk of Wilms tumor: TTC (adjusted OR=22.58, 95% CI=2.68-190.04, *P*=0.004), TCT (adjusted OR=1.56, 95% CI=1.04-2.34, *P*=0.031), CCT (adjusted OR=15.22, 95% CI=1.69-136.97, *P*=0.015) and CCC (adjusted OR=1.46, 95% CI=1.09-1.97, *P*=0.012).

## Discussion

In this study with 183 cases and 603 controls, significant association was found between the rs7132224 A>G polymorphism and Wilms tumor risk. As far as we know, the current study is the first investigation regarding the association of *RAN* and* RANBP2* gene polymorphisms with Wilms tumor susceptibility.

Previous researches have proved that *RAN* is involved in multiple cellular processes, including nucleo-cytoplasmic transport, mitotic spindle organization and the formation of nuclear envelope 28-30. RAN has been shown to be implicated in carcinomas, such as breast cancer and lung cancer, by regulating cell adhesion, migration and invasion 31. Several researches illustrated that RAN-inhibitory peptide-loaded PEG-PLGA NPs have great potential in curbing cancer through limiting the formation of active forms of RAN 32-34. In addition, the expression level of *RAN* was significantly altered in glioblastoma, pancreatic cancer, neuroblastoma, melanoma 35-40.

Recently, there is a growing list of *RAN* binding proteins, which are molecular targets for inhibiting RAN signaling pathways of in cancer cells, and provide a neoteric approach to the treatment of cancers 27, 41. RANBP2 known to regulate numerous cellular activities, is reported has an indivisible association with nasopharyngeal carcinoma through interacting with *Epac1* 42. In addition, some investigations have confirmed that RANBP2 is a tumor suppressor 43-45. Horio et al. found that the transcription of *RANBP2* was significantly higher in small cell lung cancer than in other types of lung cancers 46. Furthermore, RANBP2 was verified to be upregulated in more than half of the multiple myeloma cases^47^.

We conducted this study to explore the association of *RAN* and* RANBP2* gene polymorphisms with Wilms tumor susceptibility has not yet been explored. Compared to *RAN* rs7132224 AA genotype, AG/GG genotypes were found significantly correlated with the risk of Wilms tumor. However, we failed to significant association for other three gene polymorphisms. An early study by Ying et al. indicated that the CC genotype of *RAN* rs14035 significantly increased the venture of gastric cancer 48. And the *RAN* rs14035 CT heterozygote was a protective factor against colorectal cancer for Korean male 49. However, we failed to find significant association between the rs14035 polymorphism and Wilms tumor, which could be caused by the small sample size. We also testified that carriers of 1-3 risk genotypes were more susceptible to Wilms tumor than those without risk genotype. The stratified analysis exhibited that rs56109543 CT/TT and rs7132224 AG/GG increase the risk of Wilms tumor in children aged ≤18 months. and Carriers of 1-3 risk genotypes have a significant increased susceptibility of Wilms tumor in children aged ≤18 months and male. What's more, the rs2462788 CT/TT in *RANBP2* gene increased the chance of Wilms tumor in children aged ≤18 months, male and clinical stage I+II Wilms tumor. So far, no studies have shown a link of *RAN* rs56109543, *RAN* rs7132224 and *RANBP2* rs2462788 with other tumors, which required more researches.

Although our study is the first investigation about the association of *RAN/RANBP2* gene SNPs with Wilms tumor risk, several flaws should be noted. First, the sample size of our study was relatively small. The significant results could be just chance findings, and our results need to be verified by independent cohorts. Secondly, recruitment of participants only from Wenzhou and Guangzhou may cause selection bias; thus the results cannot fully represent other population. Thirdly, mechanisms of how the SNPs modify Wilms tumor susceptibility should be studied. Finally, we only explored the effects of genes polymorphisms on tumor susceptibility, but not on tumor development and prognosis.

In conclusion, our data provide evidence that *RAN* gene rs7132224 A>G polymorphism is significantly associated with increased Wilms tumor susceptibility. More comprehensive researches with lager sample size were warranted to validate the association between *RAN/RANBP2* gene polymorphisms and Wilms tumor risk.

## Supplementary Material

Supplementary figures and tables.

## Figures and Tables

**Table 1:** Association between *RAN/RANBP2* gene polymorphisms and Wilms tumor susceptibility

Genotype	Cases (N=183)	Controls (N=603)	*P*^ a^	Crude OR (95% CI)	*P*	Adjusted OR (95% CI) ^b^	*P*^ b^
***RAN* rs56109543 C>T (HWE=0.258)**							
CC	117 (63.93)	430 (71.31)		1.00		1.00	
CT	59 (32.24)	154 (25.54)		1.41 (0.98-2.02)	0.065	1.41 (0.98-2.02)	0.065
TT	7 (3.83)	19 (3.15)		1.35 (0.56-3.30)	0.505	1.35 (0.56-3.30)	0.504
Additive			0.164	1.31 (0.97-1.75)	0.077	1.30 (0.97-1.75)	0.077
Dominant	66 (36.07)	173 (28.69)	0.058	1.40 (0.99-1.99)	0.058	1.40 (0.99-1.99)	0.058
Recessive	176 (96.17)	584 (96.85)	0.655	1.22 (0.51-2.96)	0.656	1.22 (0.51-2.96)	0.655
***RAN* rs7132224 A>G (HWE=0.231)**							
AA	87 (47.54)	337 (55.89)		1.00		1.00	
AG	78 (42.62)	220 (36.48)		1.37 (0.97-1.95)	0.075	1.38 (0.97-1.95)	0.074
GG	18 (9.84)	46 (7.63)		1.52 (0.84-2.75)	0.170	1.52 (0.84-2.75)	0.168
Additive			0.132	1.28 (1.00-1.65)	0.052	1.29 (1.00-1.65)	0.051
Dominant	96 (52.46)	266 (44.11)	0.047	**1.40 (1.003-1.95)**	**0.048**	**1.40 (1.01-1.95)**	**0.047**
Recessive	165 (90.16)	557 (92.37)	0.339	1.32 (0.75-2.34)	0.340	1.32 (0.75-2.34)	0.338
***RAN* rs14035 C>T (HWE=0.389)**							
CC	113 (61.75)	403 (66.83)		1.00		1.00	
CT	63 (34.43)	176 (29.19)		1.28 (0.90-1.82)	0.178	1.28 (0.89-1.82)	0.179
TT	7 (3.83)	24 (3.98)		1.04 (0.44-2.48)	0.929	1.04 (0.44-2.47)	0.937
Additive			0.401	1.17 (0.87-1.55)	0.299	1.16 (0.87-1.55)	0.302
Dominant	70 (38.25)	200 (33.17)	0.205	1.25 (0.89-1.76)	0.205	1.25 (0.89-1.76)	0.207
Recessive	176 (96.17)	579 (96.02)	0.925	0.96 (0.41-2.27)	0.926	0.96 (0.41-2.26)	0.917
***RANBP2* rs2462788 C>T (HWE=0.298)**							
CC	163 (89.07)	554 (91.87)		1.00		1.00	
CT	20 (10.93)	49 (8.13)		1.39 (0.80-2.40)	0.242	1.39 (0.80-2.41)	0.240
TT	0 (0.00)	0 (0.00)		/	/	/	/
Additive			0.241	1.39 (0.80-2.40)	0.242	1.39 (0.80-2.41)	0.240
Dominant	20 (10.93)	49 (8.13)	0.241	1.39 (0.80-2.40)	0.242	1.39 (0.80-2.41)	0.240
**Combined effect of risk genotypes for *RAN ^c^***							
0	84 (45.90)	336 (55.72)		1.00		1.00	
1	28 (15.30)	68 (11.28)		1.65 (1.00-2.72)	0.051	1.65 (1.00-2.73)	0.050
2	9 (4.92)	26 (4.31)		1.39 (0.63-3.07)	0.422	1.39 (0.63-3.09)	0.415
3	62 (33.88)	173 (28.69)		1.43 (0.98-2.09)	0.060	1.43 (0.99-2.09)	0.060
0	84 (45.90)	336 (55.72)		1.00		1.00	
1-3	99 (54.10)	267 (44.28)	0.020	**1.48 (1.06-2.07)**	**0.020**	**1.49 (1.07-2.07)**	**0.020**

OR, odds ratio; CI, confidence interval; HWE, Hardy-Weinberg equilibrium. ^a^χ^2^ test for genotype distributions between Wilms tumor patients and controls. ^b^Adjusted for age and gender. ^c^ Risk genotype were with rs56109543 CT/TT, rs7132224 AG/GG and rs14035 CT/TT.

**Table 2:** Stratification analysis of* RAN* polymorphisms with Wilms tumor susceptibility

Variables	rs56109543 (cases/controls)		AOR (95% CI) ^a^	*P* ^a^	rs7132224 (cases/controls)		AOR (95% CI) ^a^	*P* ^a^	Risk genotypes (cases/controls)		AOR (95% CI) ^a^	*P* ^a^
	CC	CT/TT			AA	AG/GG			0	1-3		
**Age, month**												
≤18	46/201	30/67	**1.98 (1.15-3.38)**	**0.013**	27/161	49/107	**2.80 (1.64-4.77)**	**0.0002**	27/161	49/107	**2.80 (1.64-4.77)**	**0.0002**
>18	71/229	36/106	1.10 (0.69-1.74)	0.694	60/176	47/159	0.86 (0.56-1.34)	0.510	57/175	50/160	0.95 (0.62-1.48)	0.830
**Gender**												
Female	52/189	29/78	1.35 (0.80-2.28)	0.263	38/141	43/126	1.27 (0.77-2.09)	0.350	36/140	45/127	1.38 (0.84-2.27)	0.207
Male	65/241	37/95	1.45 (0.91-2.31)	0.123	49/196	53/140	1.52 (0.97-2.36)	0.067	48/196	54/140	**1.57 (1.01-2.46)**	**0.046**
**Clinical stages**												
I+II	43/430	24/173	1.39 (0.82-2.36)	0.226	30/337	37/266	1.56 (0.94-2.61)	0.086	30/336	37/267	1.56 (0.93-2.59)	0.090
III+IV	65/430	33/173	1.26 (0.80-1.98)	0.328	51/337	47/266	1.17 (0.76-1.80)	0.478	48/336	50/267	1.30 (0.85-2.00)	0.226

AOR, adjusted odds ratio; CI, confidence interval. ^a^ Adjusted for age and gender, without the corresponding stratify factor.

**Table 3:** Stratification analysis of *RANBP2* rs2462788 C>T polymorphism with Wilms tumor risk

Variables	rs2462788 (cases/controls)		OR (95% CI)	*P*	AOR (95% CI) ^a^	*P* ^a^
	CC	CT/TT				
**Age, month**						
≤18	63/247	13/21	**2.43 (1.15-5.11)**	**0.020**	**2.43 (1.15-5.12)**	**0.020**
>18	100/307	7/28	0.77 (0.33-1.81)	0.546	0.77 (0.33-1.82)	0.556
**Gender**						
Females	76/243	5/24	0.67 (0.25-1.81)	0.425	0.67 (0.25-1.81)	0.429
Males	87/311	15/25	**2.15 (1.08-4.25)**	**0.029**	**2.15 (1.08-4.25)**	**0.029**
**Clinical stages**						
I+II	54/554	13/49	**2.72 (1.39-5.33)**	**0.004**	**2.77 (1.41-5.46)**	**0.003**
III+IV	93/554	5/49	0.61 (0.24-1.57)	0.302	0.62 (0.24-1.59)	0.317

OR, odds ratio; CI, confidence interval; AOR, adjusted odds ratio. ^a^Adjusted for age and gender, without the corresponding stratify factor.

**Table 4:** The frequency of inferred haplotypes of *RAN* gene based on observed genotypes and their association with the risk of Wilms tumor

Haplotypes^a^	Cases (n=366)	Controls (n=1206)	Crude OR (95% CI)	*P*	AOR (95% CI)^b^	*P* ^b^
TTT	231 (63.11)	872 (72.31)	1.00		1.00	
TTC	6 (1.64)	1 (0.08)	**22.65 (2.71-189.07)**	**0.004**	**22.58 (2.68-190.04)**	**0.004**
TCT	38 (10.38)	92 (7.63)	**1.56 (1.04-2.34)**	**0.032**	**1.56 (1.04-2.34)**	**0.031**
TCC	7 (1.91)	36 (2.99)	0.73 (0.32-1.67)	0.461	0.74 (0.32-1.68)	0.465
CTT	1 (0.27)	0 (0.00)	/	/	/	/
CTC	0 (0.00)	0 (0.00)	/	/	/	/
CCT	4 (1.09)	1 (0.08)	**15.10 (1.68-135.74)**	**0.015**	**15.22 (1.69-136.97)**	**0.015**
CCC	79 (21.58)	204 (16.92)	**1.46 (1.09-1.97)**	**0.012**	**1.46 (1.09-1.97)**	**0.012**

OR, odds ratio; CI, confidence interval; AOR, adjusted odds ratio.^ a^ The haplotypes order were rs56109543, rs7132224 and rs14035. ^b^ Obtained in logistic regression models with adjustment for age and gender.

## Abbreviations

HWE: Hardy-Weinberg equilibrium
OR: odds ratio
CI: confidence interval
