# Supplementary material for: The association of RAN and RANBP2 gene polymerphisms with Wilms tumor risk in Chinese children
Source: J Cancer. 2020 Jan 1;11(4):804–9. doi: 10.7150/jca.36651 (PMC6959007; doi:10.7150/jca.36651)
Supplement: Supplementary file 1 — Supplementary figures and tables. [file jcav11p0804s1.pdf]

Supplemental Table 1. Frequency distribution of selected variables for Wilms tumor patients and cancer-free controls

Variables	Cases (N=183)		Controls (N=603)		P^{a}
	No.	%	No.	%	
Age range, month	1-144		0.07-156		0.486
Mean ± SD	29.64 ± 25.71		29.00 ± 24.00		
≤18	76	41.53	268	44.44	
>18	107	58.47	335	55.56	
Gender					0.997
Female	81	44.26	267	44.28	
Male	102	55.74	336	55.72	
Clinical stages					
I	12	6.56			
II	55	30.05			
III	58	31.69			
IV	40	21.86			
NA	18	9.84			

SD, standard deviation; NA, not available.

[a] Two-sided χ^2 test for distributions between Wilms tumor patients and cancer-free controls.
